# One Year Sustainability of Risk Factor Change from a 9-Week Workplace Intervention

**DOI:** 10.1155/2009/569104

**Published:** 2010-02-10

**Authors:** Elaine C. Rush, Michelle B. Cumin, Lela Migriauli, Lynnette R. Ferguson, Lindsay D. Plank

**Affiliations:** ^1^School of Sport and Recreation, Faculty of Health and Environmental Science, Auckland University of Technology, Private Bag 92006, Auckland 1142, New Zealand; ^2^Discipline of Nutrition, Faculty of Medical and Health Sciences, University of Auckland, Auckland 1142, New Zealand; ^3^Department of Surgery, Faculty of Medical and Health Sciences, University of Auckland, Auckland 1142, New Zealand

## Abstract

We examined the effect of a 9-week diet and physical activity intervention provided in the workplace by a group education session where personal dietary and physical activity goals were proposed. Measurements of anthropometry, fasting blood lipids, glucose and insulin, assays for antioxidant activity (AOA) and questionnaires were completed at 0, 3, 6, 9, and 12 weeks in 50 healthy workers (50% male, mean age 46y). Followup measurements in 39 (56% male) were possible at 52 weeks. At week 3 a group dietary and physical activity “motivational seminar” was held. At week 6, half the group were supplied daily kiwifruit for 3 weeks with cross over at week 9 until week 12. Compared to baseline, lipid, glucose, insulin and AOA measurements were improved at 12 and 52 weeks. Body measurements did not change. Group diet and physical activity advice reinforced over 9 weeks is associated with a sustained improvement in cardiovascular risk factors at 52 weeks.

## 1. Introduction

Diet and physical activity are major modifiable factors [1] that help prevent or delay lifestyle-related chronic diseases such as cardiovascular disease (CVD), hypertension, type 2 diabetes mellitus and cancer. The prevalence of “chronic lifestyle disease” rapidly increases as a population ages, yet it is calculated that at least 80% of CVD, stroke and type 2 diabetes, and 40% of cancer could be avoided through healthy diet, regular physical activity, and not using tobacco [2, 3]. The Global Burden of Disease Study [4] ranked 25 major risk factors for death and disability in the world. High blood pressure was ranked third, obesity fourth, high blood cholesterol seventh, low fruit and vegetable intake twelfth, and physical inactivity ranked fourteenth [5]. Research supports the claim that a diet high in fruit, vegetables, whole grains, and low in saturated fat, sugar, and salt with increased physical activity can reduce the risk of CVD and generally improve health, blood glucose concentrations, and lipid profile [6]. 

The pathophysiology associated with the development of each lifestyle disease differs and mechanisms by which diet and physical activity reduce risk are complex and related to societal and environmental factors[7]. At a physiological level beta cell and endothelial dysfunction are related to oxidative stress induced by the reactive species (RS) produced by the metabolic processes of the body [8, 9]. An oxidant-antioxidant balance [6] may be improved by consuming a healthier diet including a variety of fruits, vegetables, whole grains, and oily fish because they contain a complex mixture of natural antioxidant micronutrients that are associated with the long term outcome of disease prevention [9]. One readily available fruit that has a high level of antioxidants is kiwifruit which has been shown to protect against oxidative DNA damage [10]. We have previously shown that the provision of kiwifruit for three weeks improved laxation [11]. Further investigation of the short-term effects of kiwifruit on oxidant status and risk factors for chronic disease is warranted.

Increased physical activity and dietary change at the individual level often requires intensive one-to-one advice, and the changes may reverse after the intervention stops [12]. The workplace is a natural setting to work with groups, where collegial and social support for behavioural change can be provided and for putting in place sustainable environmental change [13]. The workplace setting allows provision and distribution of a “prescribed” fruit. 

The overall purpose of this study was to assess, after a nine-week group intervention with follow-up at one year, the effects of relatively small integrated lifestyle changes such as a healthier diet (more fruit and vegetables, whole grains and oily fish) and increased physical activity on risk factors including antioxidant status. We hypothesised that a workplace group diet and activity intervention would reduce plasma glucose and insulin, improve fasting lipid profile and increase antioxidant activity (AOA). A second hypothesis was that the addition of kiwifruit to the diet for three weeks would improve plasma glucose, lipid concentrations, and AOA.

## 2. Methods

**Figure 1:**
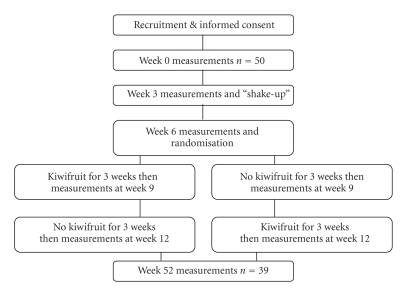
Study design.

This study was a 12 weeks longitudinal group intervention study with a followup after 52 weeks (Figure 1). Staff from the Auckland University of Technology aged 30 years or more were recruited by advertisement and personal contact. It was expected that in this age range, risk factors for lifestyle diseases would be likely to be found. Exclusion criteria were type 2 diabetes mellitus, pregnancy, known hypertension, or use of medication for elevated cholesterol. Fifty (25 male, 25 female) healthy subjects, mean age 46 years, completed all measurements at 0, 3, 6, 9, and 12 weeks. Thirty eight were self-identified as New Zealand European, 5 as British, 3 as Maori, 1 as Tongan, 2 as Samoan, and 1 as Indian. Thirty-nine subjects presented for a sixth set of measurements at 52 weeks. Of the 11 who did not present, 3 were pregnant, 1 had left the place of employment, 2 were on leave, 2 declined to participate and 3 agreed to participate but did not present. Ethical approval was granted by the AUT ethics committee, Ref 02/08.

Food frequency and physical activity questionnaires, designed to ask questions about current practice around the dietary and physical activity goals of the intervention, were completed at each time point. Measurements of anthropometry (skinfolds, waist girths, weight and height following standard techniques), blood pressure and fasting plasma lipids, glucose, insulin and antioxidant status were made at each time point. DNA fragility was measured by comet assay in a subgroup, and these results have been reported elsewhere [14].

Fasting venous blood samples were collected by a trained phlebotomist. Fresh plasma was analyzed for glucose, lipids and insulin. Samples for antioxidant measurement were put on ice in EDTA tubes and centrifuged within 10 minutes at 2500 rpm for 7 minutes. Plasma was stored at −85°C until analysis. Glucose was measured by the Roche-Hitachi glucose oxidase method, insulin by the Abbot IMx Insulin assay method, total and LDL cholesterol by the standard Roche-Hitachi methodology, and HDL cholesterol by direct assay. These measurements were made at the Diagnostic Medlab (Ltd) laboratory which is IANZ accredited. The homeostasis model assessment (HOMA) [15] calculation using a DOS programme supplied by Jonathan Levy was utilized to measure beta-cell function (HOMA-B%) and insulin sensitivity (HOMA-S%). The ferric reducing ability of plasma (FRAP) assay [16] was utilized to measure soluble antioxidants as plasma AOA. Changes in plasma malondialdehyde (MDA), as a marker of lipid peroxidation (LP) were measured as thiobarbituric acid reactive substances (TBARS) [17]. 

Between measurements at weeks 0 and 3, the volunteers were allowed to follow their usual food and activity pattern. The three week run-in period was to measure the effect of participation in an intervention study prior to the active intervention [18]. After the 3 weeks measurements the group gathered together for motivational talk on diet and exercise, and were provided with tailored written material and pedometers to increase motivation. Details of the diet and physical activity goals and advice have been published as part of a thesis [19]. In summary, the tailored written food based dietary advice recommended daily numbers of standard servings of fruit and vegetables, low fat dairy and milk products, lean meats and cereals and wholegrains adjusted according to body size, sex and need to lose weight. The number of servings of each food group was balanced over 3 meals and 3 snacks so that the recommended diet was 50%–60% energy from carbohydrates, 15–18% energy from protein and 24%–27% energy from fat. Choices of foods with less sugar, saturated fat and salt were recommended with advice emphasising an increase in dietary fibre by increasing fruit, vegetables and whole grains and an increase in omega-3 fatty acid mainly from consumption of oily fish. Two in-house booklets were distributed. One booklet contained partially individualized dietary information, hints for improving diet and physical activity, and goal sheets to help motivate participants. The other booklet included recipes to help implement dietary changes, for example, lower fat, the use of less-used ingredients like legumes, beans, and oily fish. The activity advice was based on regular and gradual increases in physical activities, which are easy to maintain and build for a long term. 

Pedometers (Digiwalker SW-700) were issued to the participants to help motivate them to increase their physical activity. It was recommended that they aim to do a minimum of 10 000 steps per day. Participants were encouraged to maintain the recommended diet and physical activity improvements throughout the study and “for the rest of their lives!” 

The third set of measurements was made at the end of week 6. After these measurements the subjects were randomly divided into two groups. The first group (A) were provided with kiwifruit at a daily dose of one kiwifruit/30 kg body weight for three weeks while the second group (B) were asked to abstain from consumption of kiwifruit. This dose was chosen because in our study of the laxative effects of kiwifruit [11] significant changes in laxation and therefore presumably effects of increased fibre were seen in 42 elderly people after 3 weeks of treatment following this regime. Following measurement at week 9, group A abstained from kiwifruit while Group B added the kiwifruit to their diet and the measurements were repeated at week 12. At weeks 9 and 12 participants were asked to provide an indication of the goals that they had set and if they had accomplished them. After completion of these measurements, participants were given a copy of their results, a group summary which showed good outcomes, and encouragement to continue. After 52 weeks, with only three-monthly emails as updates about diet and activity, they were invited to have the measurements repeated and complete the same questionnaires.

## 3. Statistical Analysis

Two-way repeated measures ANOVA and paired *t*-tests were used to assess the effect of diet and activity advice on the biochemical markers within individuals before, during and after the intervention. Baseline for the diet and activity intervention was week 0, that is, before the run-in period where participants had some knowledge of who was in the trial. Analysis of the effects of kiwifruit was performed using the methods of Hills and Armitage [20] for crossover trials to examine treatment, period and interaction effects. Data normality was tested with the Kolmogorov-Smirnov test. Statistical analysis was carried out using SPSS v.13 (SPSS Inc, Chicago, IL). Statistical significance was set at *P* < .05. Results are expressed as mean ± standard deviation (SD) unless stated otherwise.

## 4. Results

Most participants were self-reported “health conscious” individuals and were aware of the importance of healthy diet and physical activity according to their answers to the food frequency and physical activity questionnaires at the beginning of the study. However, from the baseline measurements made at weeks 0 and 3, a variety of biological risk factors were identified. Seventy-four percent of the participants were classified as being overweight or obese using BMI cut-off values [21] and 11 women and 6 men had waist measurements [22] that indicated that their abdominal fat stores were placing them at risk of CVD and type 2 diabetes. Seventy percent of subjects had elevated total plasma cholesterol (>5.0 mmol/L) and LDL cholesterol (>3.0 mmol/L) and 40% had a total to HDL cholesterol ratio over 4.5 mmol/L. Fifteen percent of participants had a high plasma insulin level (>50 pmol/L). High blood pressures (systolic >140 mmHg and/or diastolic >90 mmHg) were found in five participants. No changes in anthropometry or blood pressure were seen over the 12-week study period (Table 1). 

Compared to baseline (week 0), total cholesterol, LDL cholesterol, and total to HDL cholesterol ratio were lower at the end of the 12-week study period (Table 2). Mean total cholesterol decreased by 0.4 mmol/L over this period (*P* < .0001) and LDL cholesterol by 0.4 mmol/L (*P* = .001) but HDL cholesterol did not change (*P* = .22). Total to HDL cholesterol ratio decreased 0.3 (*P* < .0001), a change of 8%. In females compared with males, over the 12-week study period, HDL cholesterol was higher (*P* < .001) and total to HDL cholesterol ratio lower (*P* = .002, data not shown). At week 12, HDL cholesterol was 1.37 ± 0.24 mmol/L in men and 1.58 ± 0.34 mmol/L in women (*P* = .01) while total to HDL cholesterol ratio was 4.08 ± 0.92 in men and 3.41 ± 0.88 in women (*P* = .01).

Risk factors for type 2 diabetes, *viz*, plasma insulin and glucose, beta-cell function (measured as HOMA B%), and insulin sensitivity (measured as HOMA S%) remained stable over the first 12 weeks of diet and activity intervention (Table 2). During the 12-week period, mean plasma AOA increased at each measurement point but the average increase of 116 mmol/L in plasma AOA between week 0 and week 12 (from 1220 ± 423 to 1336 ± 514 *μ*mol/L) did not reach statistical significance, (*P* = .069). 

Because a smaller change in antioxidant status was expected in those who entered the study with a high antioxidant status, the cohort was divided into those with AOA < 1200 *μ*mol/L (*n* = 26) at baseline and AOA > 1200 *μ*mol/L. A significant increase (200 *μ*mol/L, from 936 ± 182 to 1136 ± 440 *μ*mol/L, *P* = .011) was seen in the participants with lower AOA at baseline and no change observed in those with high AOA (1542 ± 80 to 1561 ± 106 *μ*mol/L, *P* = .85). Lipid peroxidation did not change between week 0 and week 12.

Analysis of the kiwifruit cross-over trial showed no significant effect of kiwifruit consumption on lipid profile or glucose and insulin measures, and therefore is not reported in detail. Compliance for kiwifruit consumption was confirmed verbally when the kiwifruit was delivered each week but also confirmed by the food frequency questionnaire.

**Figure 2:**
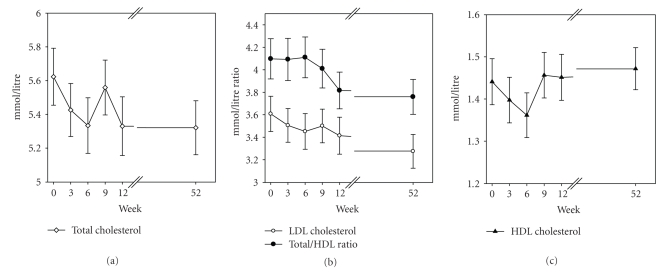
Measurements of total cholesterol (open diamond), LDL cholesterol (closed circle), the ratio of total to HDL cholesterol (open circle) and HDL cholesterol (closed triangle) over 52 weeks in 39 volunteers. Data are mean ± SEM.

In the 39 subjects (22 M, 17 F) who were measured at 52 weeks, there were no changes in anthropometric data but systolic blood pressure increased from baseline (Table 3). The 4 mmHg increase is probably not clinically meaningful. Favourable blood lipid changes seen over the 12 weeks persisted at 52 weeks (Figure 2 and Table 4); glucose and insulin decreased significantly and insulin sensitivity increased. Insulin in males was maintained (week 0, 56 ± 28; week 52, 55 ± 29 pmol/L, *P* = .87) but decreased in females (week 0, 66 ± 34; week 52, 49 ± 27 pmol/L, *P* < .0001) and insulin sensitivity increased in females only (*P* < .001). AOA increased, particularly in those who had an initial level less than 1200 *μ*mol/L.

An increase in plasma AOA was maintained by 16 (73%) of the 22 low AOA participants measured at one year (*P* = .01). Lipid peroxidation increased a little at week 52.

Assessed by food frequency questionnaire over the 52-week period, the proportion of participants consuming fruit at a rate of 2 or more portions per day increased from 47% to 62% and for oily fish 3 times a week from 14% to 42%. The number of participants reporting that they were undertaking any moderate or vigorous activity during the week increased from 36% to 56%. Associations of change in frequency of consumption of fruits, vegetables, whole grain and time spent in physical activity with changes in metabolic risk were explored but the only significant relationship was between an increase in the frequency of intake of whole grains and a reduction in total cholesterol (*r* = −0.323, *P* = .02). From week 0 to week 52 there was a significant increase in kiwifruit consumption (Wilcoxon signed rank test, *P* < .001) with 22 increasing and 3 reducing their consumption. Kiwifruit consumption from weeks 6–9 was not a valid comparison as it was supplied to only half the group.

## 5. Discussion

The overall question asked in this study was “will group advice and support to increase daily fruit, vegetable, whole grain and oily fish intake and increase physical activities improve glucose and lipid levels and antioxidant activity in the blood and therefore, decrease the risk of lifestyle diseases?”

The answer is yes. This study demonstrated that a group work-based dietary and activity intervention was associated with reduction of some biomarkers of risk for lifestyle disease.

A reduction in total cholesterol levels of 0.6 mmol/L is associated with a 24% reduction in death from ischemic heart diseases [23]. The improvement in lipid profile was one of the sustained changes in this study. Total cholesterol was reduced from 5.7 ± 1.1 to a mean 5.3 ± 1.0 mmol/L over the 12 weeks and maintained at that level at 52 weeks. One third of the participants in this study at one year decreased their total plasma cholesterol by more than 0.6 mmol/L, maintained their HDL levels and sustainably and significantly improved their total cholesterol/HDL ratio. 

This intervention also resulted in an overall significant decrease in plasma glucose, and an increase in insulin sensitivity, which must be considered an encouraging trend. Most studies looking at reduction of type 2 diabetes risks have looked at changes that are aiming to improve the metabolic control of people with type 2 diabetes or who already have impaired glucose tolerance (IGT) [24]. Given the huge increase in the prevalence of type 2 diabetes and the expected increase in incidence over the next decade, it is important to implement lifestyle changes in those who appear to be normoglycemic as well as in those who have IGT.

The changes in the blood biochemistry were not accompanied by changes in weight or waist measurement. It was not one of the aims of this study to reduce weight or body fat substantially. Anthropometric changes or prevention of weight gain take time, particularly when the objective is for the changes to be small and sustainable, rather than to make a substantial impact on body composition that could possibly compromise sustainability. 

The changes in diet and physical activities significantly increased the plasma soluble antioxidant activity during the 12-week period, and therefore potentially decreased the level of oxidative damage. As was expected, antioxidant status improved more significantly in subjects with relatively low initial AOA and maintained that elevation out to one year. 

Some fruits or vegetables are particularly rich in antioxidant vitamins and phytochemicals, and it is reasonable to suggest that there will be significant health benefits associated with regular consumption of that particular fruit or vegetable. In this regard the effect of kiwifruit on markers of oxidative stress and blood biochemistry was tested as a part of this intervention study, in a cross over trial design. Kiwifruit contains very significant amounts of antioxidants such as ascorbic acid, folate and flavonoids [25]. There are on average 1.6 grams of dietary fiber in one kiwifruit. The antioxidant properties of this fruit have been demonstrated to protect against oxidative DNA damage [10] and to stimulate DNA repair [14, 26] by promoting DNA repair enzymes. Flavonoids present in kiwifruit may also have antioxidant as well as anti-thrombotic properties. It has recently been reported that consuming 2 or 3 kiwifruit per day for 28 days reduces platelet aggregation and decreases plasma triglyceride levels [27]. Both these responses are favourable for reduction of risk factors for CVD. However, in the present study, the effect of three weeks treatment with kiwifruit on markers of oxidative stress was rather more modest than anticipated, and did not significantly affect blood biochemistry over 3 weeks. Specifically, the 3-week daily kiwifruit consumption (2-3 kiwifruit per day) resulted in a significant increase in plasma AOA, as measured by FRAP, in female but not in male participants, but did not change markers of LP measured as plasma MDA in either sex. 

This study was limited by the self-selection of participants, its relatively small sample size and incomplete follow-up of all participants. The identification of responders and non-responders to changes in antioxidant status and apparently a better response (or compliance) from women needs to be studied further. Many of the changes made by the participants of this study were not measurable, but most participants personally described changes in attitude, awareness and motivation. This study has shown that group advice in the workplace for changes in diet and physical activity is associated with favourable changes in blood biochemistry even without accompanying changes in waist and weight. Furthermore, the biochemical changes and presumably changed behaviour persisted over 52 weeks. The next step is to change the workplace environment to support the healthier food choices and opportunities for physical activity so that the healthy choice becomes the easier choice.

##  Author Disclosures 

Elaine C. Rush, Michelle B. Cumin, Lela Migriauli, Lynn R. Ferguson, Lindsay D. Plank have no conflicts of interest.

## Figures and Tables

**Table 1:** Anthropometry for 50 subjects over 12 weeks.

	Week 0		Week 3		Week 6		Week 9		Week 12		P*
	Mean	±SD	Mean	±SD	Mean	±SD	Mean	±SD	Mean	±SD	
Weight, kg	80.0	±13.2	80.1	±13.2	80.0	±13.0	80.1	±12.9	80.0	±13.1	.98
BMI, kg/m^2^	27.0	±4.6	27.1	±4.6	27.0	±4.5	27.1	±4.6	27.1	±4.5	.99
Waist, cm	91.2	±11.4	90.8	±11.4	90.5	±11.3	91.4	±11.0	91.8	±10.8	.17
Systolic, mmHg	117	±14	120	±12	117	±12	118	±12	117	±14	.55
Diastolic, mmHg	77	8	76	±9	75	±8	76	±10	76	±10	.51

*P value for changes over time by repeated measures ANOVA with sex as fixed effect (time × sex and sex effects were nonsignificant for all variables). BMI, body mass index.

**Table 2:** Blood biochemistry for 50 subjects over 12 weeks.

	Week 0		Week 3		Week 6		Week 9		Week 12		^†^ *P*
	Mean	±SD	Mean	±SD	Mean	±SD	Mean	±SD	Mean	±SD	
Total cholesterol, mmol/L	5.7	±1.1	5.5	±1.0*	5.4	±1.0*	5.5	±1.0∗	5.3	±1.0*	<.001
LDL cholesterol, mmol/L	3.6	±1.0	3.5	±0.9	3.4	±1.0*	3.4	±0.9∗	3.3	±0.9*	<.001
HDL cholesterol, mmol/L	1.47	±0.31	1.40	±0.30	1.37	±0.30	1.46	±0.32	1.47	±0.31	^‡^.19
Total cholesterol /HDL	4.03	±1.11	4.08	±1.12	4.09	±1.11	4.00	±1.08	3.74	±0.95*	^‡^ < .001
Triglyceride, mmol/L	1.3	±0.7	1.3	±0.7	1.3	±0.7	1.5	±0.9	1.2	±0.6	.59
Glucose, mmol/L	5.2	±0.5	5.1	±0.5	5.0	±0.5	5.0	±0.5	5.1	±0.5	.16
Insulin, pmol/L	56	±27	57	±29	53	±26	57	±28	54	±30	.49
HOMA B%	90	±28	93	±27	91	±27	97	±27	89	±27	.79
HOMA S%	121	±55	115	±66	122	±53	119	±54	120	±45	.12
AOA (*μ*mol/L)	1221	±423	1260	±425	1293	±479	1338	±451*	1336	±514	.03
TBAR (mmol/L)	1.79	±0.25	1.77	±0.25	1.78	±0.23	1.79	±0.25	1.80	±0.25	^‡^.37

*Significantly different to week 0; ^†^ *P* value for changes over time by repeated measures ANOVA with sex as fixed effect (time × sex effects were nonsignificant for all variables); ^‡^women significantly different to men, HOMA homeostatic model assessment, AOA antioxidant activity, TBAR thiobarbituric acid reactive substances.

**Table 3:** Anthropometry at baseline (week 0) and follow-up (week 52) for 39 subjects.

	Week 0		^†^ *P*		^†^ *P*
	Mean	±SD	Mean	±SD	
Weight, kg	80.0	±14.1	79.9	±14.4	.98
BMI, kg/m^2^	27.0	±4.6	27.0	±4.8	.96
Waist, cm	90.8	±11.5	91.0	±10.8	^‡^.69
Systolic, mmHg	118	±14	122	±13	<.007
Diastolic, mmHg	77	±8	77	±9	.75

^†^ *P* value for changes over time by repeated measures ANOVA with sex as fixed effect (time × sex effects were nonsignificant for all variables)

^‡^women significantly different to men.

**Table 4:** Blood biochemistry at baseline (week 0) and follow-up (week 52) for 39 subjects.

	Week 0		Week 52		^†^ *P*		
	Mean	±SD	Mean	±SD	time	sex	time × sex
Total cholesterol, mmol/L	5.6	±1.1	5.3	±1.0	<.001	.20	.82
LDL cholesterol, mmol/L	3.6	±1.0	3.2	±0.9	<.001	.07	.91
HDL cholesterol, mmol/L	1.44	±0.34	1.47	±0.31	.26	.06	.50
Total cholesterol /HDL	4.10	±1.11	3.76	±0.97	<.001	.001	.29
Triglyceride, mmol/L	1.2	±0.5	1.3	±0.5	.68	.05	.06
Glucose, mmol/L	5.2	±0.4	5.0	±0.4	.01	.39	.59
Insulin, pmol/L	59	±29	50	±26	.005	.52	.023
HOMA B%	93	±30	90	±27	.25	.28	.09
HOMA S%	110	±48	128	±51	.02	.76	.05
AOA (*μ*mol/L)	1213	±429	1320	±372	.03	.36	.81
TBAR (mmol/L)	1.79	±0.24	1.83	±0.25	.06	.08	.87

^†^ *P* values for repeated measures ANOVA with sex as fixed effect: HOMA homeostatic model assessment, AOA antioxidant activity, TBAR thiobarbituric acid reactive substances.
